# Overexpression of EMMPRIN is associated with lymph node metastasis and advanced stage of non-small cell lung cancer: a retrospective study

**DOI:** 10.1186/s12890-017-0540-1

**Published:** 2017-12-28

**Authors:** Bing Liu, Zhaohui Wan, Baowei Sheng, Yong Lin, Tian Fu, Qingdi Zeng, Congcong Qi

**Affiliations:** 1 Department of Respiratory Medicine, Jining NO.1 People’s Hospital, Jiankang Road, Jining City, Shandong Province 272000 People’s Republic of China; 2 Department of General Practice, Jining NO.1 People’s Hospital, Jining, China

**Keywords:** Non-small cell lung cancer, NSCLC, EMMPRIN, Lymphatic metastasis, Biomarker

## Abstract

**Background:**

Previous studies show that overexpression of EMMPRIN involved in the malignant biological behavior of tumors. This investigation was to disclose the expression status of EMMPRIN in non-small cell lung cancer (NSCLC) and its clinical value for the diagnosis of NSCLC.

**Methods:**

The expression of EMMPRIN was examined using immunohistochemistry and enzyme-linked immunosorbent assay. The clinical value of EMMPRIN was evaluated by drawing a receiver operating characteristic (ROC) curve.

**Results:**

NSCLC tissues and serum exhibited higher expression levels of EMMPRIN than the normal control (*p* < 0.05), and the expression of the EMMPRIN was significantly associated with lymphatic invasion and advanced stage of NSCLC (*p* < 0.05). ROC curve suggested that the threshold level of serum EMMPRIN for distinguishing NSCLC from control group was 80.3 pg/mL, and displayed a sensitivity of 97.22% and a specificity of 95%. And higher EMMPRIN expression in serum and tissues appeared to be risk factors for NSCLC development (risk ratio =1.56 and 1.1).

**Conclusion:**

Overexpression of EMMPRIN was associated with lymphatic metastasis and advanced stage of NSCLC and test of serum EMMPRIN contributes to the NSCLC diagnosis.

## Background

Lung cancer is one of the leading causes of deaths globally, especially in developing countries like China. One recent investigation shows that there will be about 733,000 newly diagnosed lung cancer cases in 2015 in China and about 610,000 Chinese will die from this disease [1]. Non-small cell lung cancer (NSCLC) accounts for the majority of lung cancer (75%) in clinic, and most of them are lung squamous cell carcinoma (LSCC) and lung adenocarcinoma (LAC). People believe that early diagnosis of lung cancer play an important role in reducing of morbidity and mortality [2]. This is a consensus that the change levels of some molecules contribute to the diagnosis, treatment and prognosis of cancers [3–5]. And some valuable tumor biomarkers derived from these molecules are considered to be beneficial for molecular typing and individualized therapy [5].

Extracellular matrix metalloproteinase inducer (EMMPRIN), other names now include CD147, basigin, and HAb18G, which is originally isolated from the LX-1 human lung cancer cell line and is now considered to be a transmembrane glycoprotein [6]. EMMPRIN is a 269 aa TM protein with a glycosylated molecular weight with an approximate range of 43–66 kDa [7]. Now EMMPRIN in normal cell function and other disease states has been investigated, especially in malignant tumors. In a series of tumors, including liver, breast, colon, prostate and esophageal cancer, EMMPRIN often exhibits a high expression [7]. Recent reports have indicated that the expressions of EMMPRIN correlate with poor clinical factors and outcomes in lung cancer but some studies have inconsistent conclusions [8]. Therefore, we conducted this study for analyzing the relationship between EMMPRIN and clinical features of NSCLC, and disclosed the clinical significance and diagnostic value of EMMPRIN expression in NSCLC.

## Methods

### Patients

From January 2015 to December 2016, the blood samples from 72 patients with NSCLC at the Jining NO.1 People’s Hospital, Jining, China were collected for this retrospective study. Over the same period, the blood samples of 60 individuals for control group (receiving strict physical examination) were gathered as normal control (56 ± 3.1 years) from the hospitals mentioned above. All patients were not received radio/chemotherapy before collecting specimens and were grouped according to the tumor-node-metastasis (TNM) classification (IASLC, Eighth edition, 2016) [9]. The lung cancer tissues from 55 of 72 patients who underwent surgical resection were collected for studying on EMMPRIN expression in tissues. Meanwhile the matched adjacent non-malignant tissues were also collected as the normal control tissues, which was at least 3 cm away from the edge of lung cancer mass. The patients were grouped according to different clinical features, which are showed in Table 1.

**Table 1:** Clinico-pathological features of patients (*n*=72)

Items	Characteristics	Lung cancer patients (*N*=72)	Healthy individuals (*N*=60)
Gender			
	Male	46(63.9%)	38(63.3%)
	Female	26(36.1%)	22(36.7%)
Ages			
	<60	31(43.06%)	36(60%)
	≥60	41(56.94%)	24(40%)
Smoking			
	Yes	28(38.9%)	19 (31.7%)
	No	44(61.1%)	41(68.3%)
Classification of pathology			
	LAC	33(45.83%)	
	LSCC	39(54.17%)	
Differentiation degree			
	Poorly differentiated	24(33.3%)	
	Moderately differentiated	19(26.4%)	
	Well-differentiated	29(40.3%)	
Clinical staging			
	IIA - IIIA	35(48.6%)	
	IIIB - IV	37(51.4%)	
Lymphatic metastasis			
	N0 - N1	26(63.9%)	
	N2 - N3	46(36.1%)	

NSCLC, non-small cell lung cancer; LAC, lung adenocarcinoma; LSCC, lung squamous cell carcinoma; N, the grade of lymphatic invasion

The study was approved by Research Ethics Committee of Jining NO.1 People’s Hospital, Jining, China. Each specimen was collected after the patient's permission, while patients signed informed consent. The entire process of collecting the specimen is ethical and regulated by the approved body. We confirmed that all methods were performed in accordance with the relevant guidelines and regulations.

### Production and preservation of tissue microarray (TMA)

Before constructing the TMA, we carefully selected the representative tissue areas of lung cancer and marked. We used a tissue array instrument (Beecher Instruments, Manual Tissue Arrayer, USA) to construct the TMA. The tissue cores from donor tissue block were took out using a thin-walled needle of diameter of approximately 2.0 mm, and were precisely inserted a recipient block according to the selection number. TMA tissue blocks were sliced, baked in an oven, and stored at 4 °C.

### Treatment of blood samples

Blood samples were collected by a method of conventional venous blood collection. After collection, the blood samples were immediately mixed to avoid the generation of air bubbles. Then, the blood was centrifuged at 3000 rpm for 10 min, and the serum was carefully isolated and frozen at -20°C. The cryopreserved serum is taken out before testing and thawed at room temperature (ensuring that the sample is well thawed evenly).

### Immunohistochemistry (IHC)

The expression of EMMPRIN in tissues was determined using a Strept Actividin-Biotin Complex of IHC techniques (SABC kit, Bostere Biotech Company, Wuhan, China) according to the protocol of the SABC kit. A rabbit anti-human EMMPRIN monoclonal antibody was used as primary antibody with a dilution concentration of 1:50. The positive staining sections provided by antibody kit were served as a positive control and the first antibody replaced by the same volume of PBS was considered as a negative control. Immunostaining was blindly evaluated according to previous method reported [9]. The staining was scored separately as follows: 0 for no staining in tumour cells; 1 for moderate intensity; and 2 for intense staining of more than 75% of tumour cells; >1 was considered to be a positive result.

### Enzyme-linked immunosorbent assay (ELISA)

The EMMPRIN level of serum was measured by ELISA Kit (Shanghai Senwei Technology Industrial Co., Ltd; Shanghai, China) strictly following the testing procedures designed by the manufacturer. Samples and standards were added to the wells, and then the antibody mixture was added. After the incubation, each well was carefully washed to remove unbound material. Followed by the addition of TMB substrate, and catalyzed by HRP, resulting a blue. The final reaction is terminated by adding a stop solution, resulting in a change from blue to yellow color. The color reaction was measured by a photometer at a wavelength of 450 nm. Standard curves were made according to the concentration of the standard sample and corresponding value of optical density of each well.

### Statistical analysis

The data on the EMMPRIN expression in tissues were tested using the χ2, Fisher's exact, and McNemar test. The data on the serum level of EMMPRIN were analyzed using the Student’s *T*-test (matched samples) and One-WAY ANOVA (single factor analysis). To assess the diagnostic potential of EMMPRIN in serum for NSCLC, a ROC analysis was performed. All tests were two-sided, and p-values <0.05 were considered to be statistically significant. The statistical analysis was finished using the SPSS 19.0 software package (SPSS Institute, Chicago, USA).

## Results

### Expressions of EMMPRIN was up-regulated in NSCLC

**Fig. 1:**
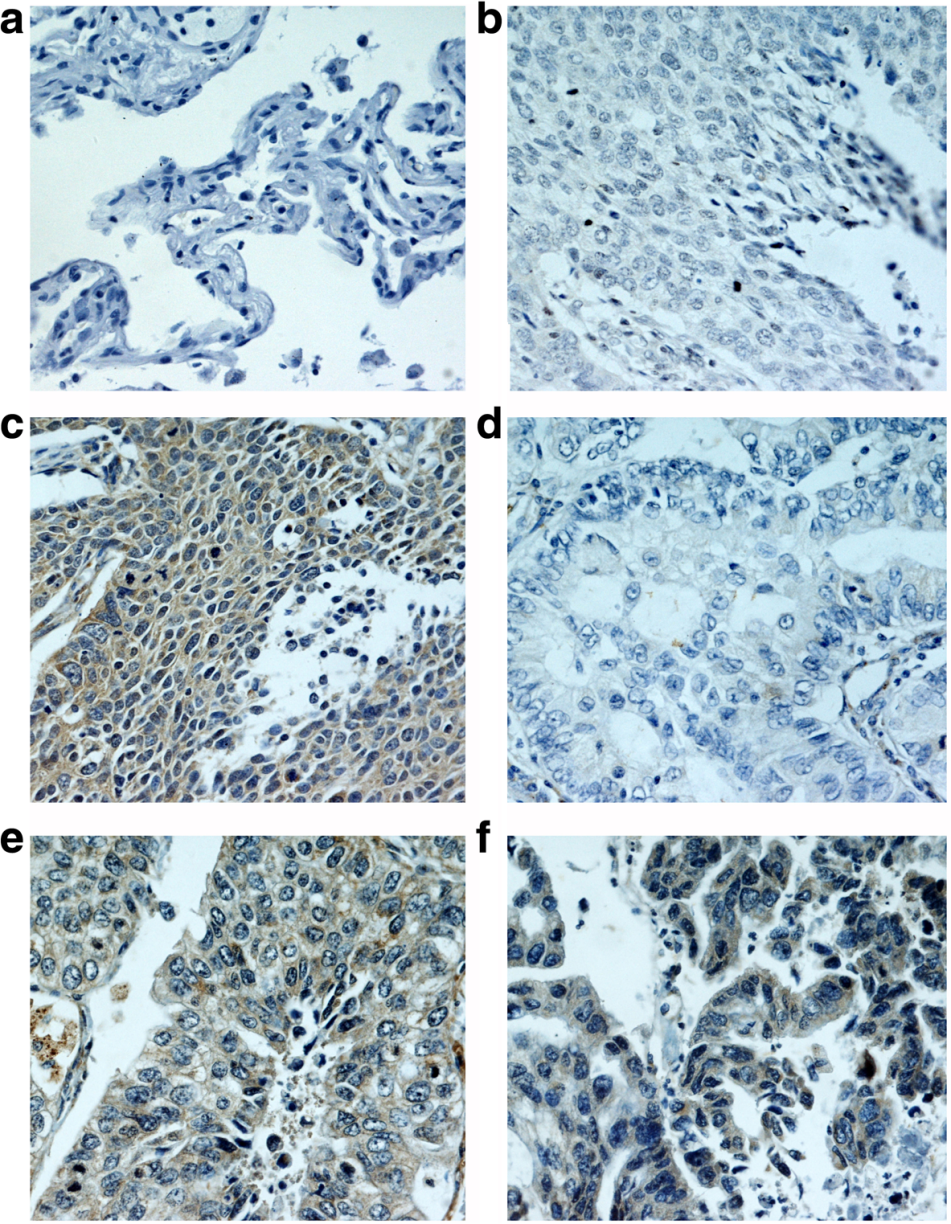
IHC analysis of EMMPRIN in NSCLC and normal tissues (IHC×400) **a**. No staining of EMMPRIN in normal tissues; **b**. No staining of EMMPRIN in well differentiated LSCC; **c**. Intensive staining of EMMPRIN in poorly differentiated LSCC; **d**. No staining of EMMPRIN in well differentiated LAC; **e**. Moderate staining of EMMPRIN in moderately differentiated LAC; **f**. Intensive staining of EMMPRIN in poorly differentiated LAC; NSCLC, non-small cell lung cancer; LAC, lung adenocarcinoma; LSCC, lung squamous cell carcinoma

EMMPRIN protein is mainly expressed in the cytoplasm and cell membrane of NSCLC (Fig. 1a–f). The statistical results showed that EMMPRIN was highly expressed in 29 (52.7%) of the 55 NSCLC tissues, whereas it was lowly expressed in 14 (25.5%) of the 55 matched adjacent non-malignant tissues. The up-regulation of EMMPRIN in the NSCLC tissues and the downregulation in the adjacent non-malignant tissues were observed (*p* = 0.003) (Table 2).

**Table 2:** Correlation between clinico-pathological features and the expressions of EMMPRIN in NSCLC tissues (*n*=55)

Items	Groups	N	Expressions of EMMPRIN in lung tissues			
			Negative (%)	Positive (%)	χ^2^ value	*P* value
Resourse						
	Normal	55	41(74.5)	14(25.5)	8.591	0.003
	NSCLC	55	26(47.3)	29(52.7)		
Gender						
	Male	36	19(52.8)	17(47.2)	1.267	0.260
	Female	19	7(36.8)	12(63.2)		
Ages						
	<60	27	13(48.1)	14(51.9)	0.016	0.898
	≥60	28	13(46.4)	15(53.6)		
Smoking						
	Yes	21	13(61.9)	8(38.1)	2.918	0.088
	No	34	13(38.2)	21(61.8)		
Histology						
	LAC	23	7(30.4)	16(69.6)	4.496	0.034
	LSCC	32	19(59.4)	13(40.6)		
Pathological grade						
	Poorly	17	2(11.8)	15(88.2)	20.762	<0.001
	Moderately	13	4(30.8)	9(69.2)		
	Well	25	20(80)	5(20)		
Lymphatic invasion						
	N0 - N1	26	20(76.9)	6(23.1)	17.392	<0.001
	N2 - N3	29	6(20.7)	23(79.3)		
pTNM						
	IIA-IIIA	35	24(68.6)	11(31.4)	17.517	<0.001
	IIIB	20	2(10)	18(90)		

LAC, lung adenocarcinoma; LSCC, lung squamous cell carcinoma; Smoking, pack years of smoking; T stage, tumor size; pTNM, clinical stage of lung cancer

### Over-expression of EMMPRIN correlated with histological type, poor differentiation, lymph node metastasis and advanced stage of NSCLC

As shown in Table 2, the expression of EMMPRIN did not relate to gender, age, and smoking status of NSCLC patients (*p* > 0.05). However, the expression level of EMMPRIN was up-regulated in LAC (16/23, 69.6%) (*p* = 0.034), poorly differentiated lung cancer tissues (15/17, 88.2%) (*p* < 0.001), NSCLC cases with N2-N3 of lymph node metastasis (23/29, 79.3%) (*p* < 0.001) and NSCLC tissues of stages IIIB (18/20, 90%) (*p* < 0.001), as compared with LSCC (13/32, 40.6%), well-differentiated tissues (5/25, 20%), lung cancer cases with N0-N1 of lymph node metastasis (6/26, 23.1%) and those of stages IIA-IIIA (11/35, 31.4%).

### Higher serum level of EMMPRIN was showed in NSCLC patients

In a series of 72 serum specimens of NSCLC patients and a series of 60 serum specimens of control individuals, the statistical analysis of Kolmogorov-Smirov indicated that two sets of data from test value of serum EMMPRIN showed an approximately normal distribution (*p*=0.122). Student's t test showed that high serum level of EMMPRIN was found in NSCLC patients (101.93±15.01 pg/mL), whereas it was lowly expressed in control group (59.04±10.97 pg/mL), indicating that the serum level of EMMPRIN was higher in NSCLC patients than that in control individuals *(p* < 0.001) (Table 3).

**Table 3:** Correlation between clinico-pathological parameters and the expressions of EMMPRIN in serum of NSCLC (*n*=72)

Parameter	Group	N	Expressions of EMMPRIN in serum of NSCLC			
			Value (pg/ml)	Degree of freedom	Statistical value	*P* value
Resource						
	Normal	60	59.04±10.97	130	18.411	<0.001
	NSCLC	72	101.93±15.01			
Gender						
	Male	46	100.86±15.09	70	-0.800	0.43
	Female	26	103.81±14.98			
Ages						
	<60	31	101.12±12.06	70	-0.396	0.69
	≥60	41	102.56±17.03			
Smoking						
	Yes	28	103.23±16.06	70	0.585	0.56
	No	44	101.09±14.13			
Histology						
	LAC	33	104.02±13.13	70	1.087	0.28
	LSCC	39	100.16±16.39			
Pathological grade						
	Poorly	24	105.33±11.46^★^	2	3.719	0.029
	Moderately	19	106.24±14.92^★^			
	Well	29	96.28±16.27			
Lymphatic invasion						
	N0 - N1	26	91.78±9.97	70	-4.983	<0.001
	N2 - N3	46	107.66±14.39			
pTNM						
	IIA - IIIA	35	93.92±10.45	70	-5.125	<0.001
	IIIB - IV	37	109.49±14.83			

M±SD, mean±standard deviation; LAC, lung adenocarcinoma; LSCC, lung squamous cell carcinoma; ^★^, poor and moderate differentiation compared with well differentiation; N, lymphatic metastasis; pTNM, clinical stage of lung cancer

### Serum level of EMMPRIN correlated with poor differentiation, lymph node metastasis and advanced stage of NSCLC

As shown in Table 3, High serum level of EMMPRIN was observed in poorly differentiated lung cancer patients (105.33±11.46 pg/mL) and moderately differentiated lung cancer patients (106.24±14.92 pg/mL), as compared with well differentiated lung cancer patients (96.28±16.27 pg/mL) (*p*=0.029). In addition, the serum level of EMMPRIN in NSCLC patients with N0-N1 of lymph node metastasis was 91.78±9.97 pg/mL, which is lower than those lung cancer patients with N2-N3 of lymph node metastasis (107.66±14.39 pg/mL) (*p* < 0.001). Moreover, the serum level of EMMPRIN was highly expressed in NSCLC patients of stages IIIB-IV (109.49±14.83 pg/mL) compared with those of stages IIA-IIIA (93.92±10.45 pg/mL) (*p* < 0.001). However, the serum level of EMMPRIN did not correlate to gender, age, smoking status and histology of NSCLC patients (*p* > 0.05).

### Threshold value and diagnostic accuracy of serum EMMPRIN for discerning NSCLC from control group

**Fig. 2:**
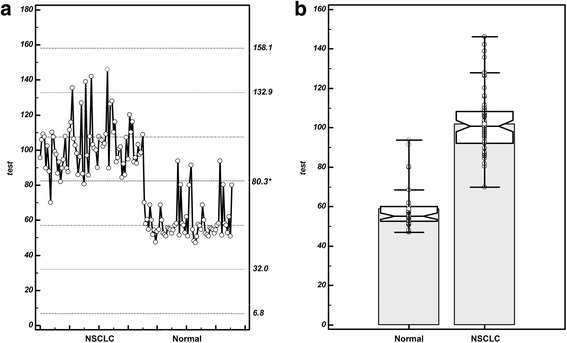
Threshold value of serum EMMPRIN for discerning NSCLC from control group **a**. Selection of cut-off value (80.3 pg/mL) of serum EMMPRIN for discerning NSCLC patients from control group, responding a sensitivity of 97.22% and specificity of 95%; **b**. The minimum value of EMMPRIN was 47.11 pg/ mL, which was observed in the control group while the maximum was 146.23 pg/mL, located in the disease group with NSCLC; NSCLC, non-small cell lung cancer

**Fig. 3:**
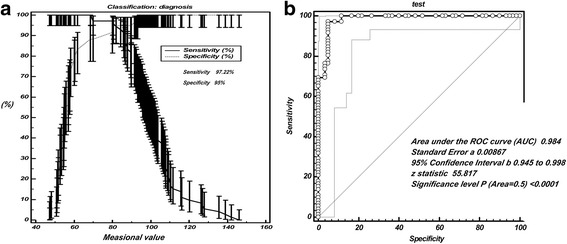
Diagnostic accuracy of serum EMMPRIN for discerning NSCLC from control group **a**. The cut-off value of EMMPRIN for distinguishing NSCLC patients from control group was finally defined as 80.3 pg/mL, responding a sensitivity of 97.22% and specificity of 95%; **b**. ROC curve analysis showed that the AUC (area under the curve) of EMMPRIN arrived at 0.984 and Z value was 55.817 (*P*<0.001); ROC, receiver operating characteristic curve; AUC, area under the curve

The cut-off value of serum EMMPRIN for discerning NSCLC was determined via ROC curve analysis [10]. As shown in Table 4, the threshold of serum EMMPRIN discerning NSCLC from control group intersected near the zone of 70 to 91.4 pg/mL, and the 95% CI (confidence interval) was from 77.4 to 99.6. The cut-off value of EMMPRIN for distinguishing NSCLC patients from control group was finally defined as 80.3 pg/mL (Fig. 2a). The minimum serum value of EMMPRIN was 47.11 pg/mL, which was in the control group while the maximum was found in the NSCLC group (146.23 pg/mL) (Fig. 2b). When 80.3 pg/mL is used as a threshold, the sensitivity of serum EMMPRIN for discerning NSCLC from normal individuals was 97.22% and specificity was 95% (Fig. 3a). ROC curve analysis showed that the AUC (area under the curve) of EMMPRIN arrived at 0.984 with 0.00867 of standard error, while 95% CI was from 0.945 to 0.998, and then Z value was 55.817 (*P* < 0.0001) (Fig. 3b).

**Table 4:** Criterion values and coordinates of the ROC curve for EMMPRIN

Criterion	Sensitivity	95% CI	Specificity	95% CI	+LR	95% CI	-LR	95% CI
>68.6	100.00	95.0 - 100.0	88.33	77.4 - 95.2	8.57	7.8 - 9.4	0.00	-
>70	97.22	90.3 - 99.7	88.33	77.4 - 95.2	8.33	7.5 - 9.2	0.031	0.007 - 0.1
>80.1	97.22	90.3 - 99.7	91.67	81.6 - 97.2	11.67	10.7 - 12.7	0.030	0.006 - 0.2
>80.3 *	97.22	90.3 - 99.7	95.00	86.1 - 99.0	19.44	18.1 - 20.9	0.029	0.005 - 0.2
>87.6	84.72	74.3 - 92.1	95.00	86.1 - 99.0	16.94	15.1 - 19.0	0.16	0.05 - 0.6
>91.4	76.39	64.9 - 85.6	96.67	88.5 - 99.6	22.92	20.0 - 26.3	0.24	0.06 - 1.0
>93.9	69.44	57.5 - 79.8	100.00	94.0 - 100.0	-	-	0.31	-

95% CI, 95% confidence; +LR, positive likelihood ratio; -LR, negative likelihood ratio; *cut-off value of EMMPRIN for distinguishing NSCLC patients from control group

### EMMPRIN expression in lung cancer tissue and serum of NSCLC patients displayed a positive correlation

When a serum value of EMMPRIN that is greater than a threshold of 80.3 pg/mL was defined as positive and a value of less than 80.3 pg/mL was defined as negative. The results showed that the positive co-expression rate of serum and tissues of EMMPRIN in NSCLC was 50.9% (28/55) and the negative co-expression rate was 3.6% (2/55). Fisher’s Exact Test suggested that the difference of EMMPRIN positive rate in cancer tissue and serum was not statistically significant (*P*=0.001) (Table 5). However, the P value of *McNemar Test* was less than 0.001, suggesting that EMMPRIN expression in lung cancer tissue and serum of NSCLC patients had a correlation.

**Table 5:** Correlation of EMMPRIN expression in lung tissue and serum of NSCLC patients (*n*=55)

Items	55 cases	Tissues		Pearson Chi square	Fisher’s Exact Test^★^	*P* value	*McNemar Test* ^*#*^
		Positive (%)	Negative (%)				
Serum	Positive (%)	28(50.9)	25(45.5)	0.432	0.605	0.515	*P*<0.001
	Negative (%)	1(1.8)	2(3.6)				

^★^Statistical results suggest: 2 cells (50.0%) have expected count less than 5. The minimum expected count is 1.45; ^*♯*^McNemar Test's *P* value is less than 0.001, indicating that EMMPRIN positive rate in cancer tissue and serum were statistically correlated

### The relative risk (RR) for the over-expression of EMMPRIN in NSCLC

Pearson’s Chi square-test was employed to evaluate the risk ratio on the expressions of EMMPRIN in serum and tissues of NSCLC. The results indicated that the risk ratio value of over-expression of EMMPRIN in serum was 1.56 (*P* < 0.001) with a 95% confidence interval (CI) of 1.301 to 1.84. In addition, the risk ratio value in tissues was 1.1 (*P* < 0.001), and the 95% CI was 0.68 to 1.35. The results showed that subjects with higher EMMPRIN expression in serum and tissues implied a higher risk for NSCLC possibility (risk ratio =1.56 and 1.1) compared with subjects with lower EMMPRIN expression, and the RR of EMMPRIN expression in serum is greater than in tissues.

## Discussion

The clinical management decisions of lung cancer patients are increasingly dependent on the guidance of by prognostic and predictive markers. At present, some valuable molecular markers play an increasingly important role in the individualized treatment of tumors [11]. Most of the NSCLC are usually diagnosed before the disease reaches a late stage, resulting in a low 5-year survival rate of 20% [12]. The occurrence and development of NSCLC is involved a wide range of molecular biological changes. With the development of molecular technologies, increasingly more tumor markers have been applied in clinic [13]. Carcinoembryonic antigen (CEA), cytokeratin 19 fragments (CYFRA 21-1) and squamous cell carcinoma antigen are commonly recommended in NSCLC management [14]. EMMPRIN is encoded by a gene localized to 19p13.3, which has recently been recognized as an important modulator of tumor-stromal communication and mediates a wide range of tumor-promoting molecular events [15]. EMMPRIN is mainly known for its protease inducing function but a role in promoting tumor angiogenesis has also been demonstrated [16]. So far, the exploration on EMMPRIN has focused on basic research in vitro, and the number of studies on its expression in lung cancer is limited. In our study, the clinical significance of EMMPRIN expression in serum and tissues of NSCLC patients were evaluated.

In tissues level, our findings showed that EMMPRIN exhibited a higher expression in NSCLC than in adjacent non-malignant tissues. Therefore, we have reason to believe that there is relation between the high expression of EMMPRIN and the occurrence and development of lung cancer. We found that EMMPRIN expression was higher in LAC than in LSCC. In addition, the EMMPRIN was also highly expressed in poorly differentiated lung cancer tissues. In clinic, LAC is more aggressive and often early metastasizes to liver and brain, compared with LSCC and poorly differentiated lung cancer progresses faster. Thus, the results may suggest that there is a correlation between EMMPRIN over-expression and these malignant biological behaviors of NSCLC. According to our study, over-expression of EMMPRIN is closely related to lymph node invasion and advanced TNM staging of NSCLC. Studies have shown that metastasis of tumor cells and lymph node invasion are key factors in the progression of NSCLC. Therefore our study suggests that EMMPRIN over-expression may promote the development and progression of NSCLC. Our findings is consistent with the previous reports, which showed that compared with patients with low expression level of EMMPRIN, higher level of EMMPRIN in cancer tissues were associated with poor prognosis [15–18].

In serum level, our results showed that the serum EMMPRIN showed a remarkable elevation in NSCLC patients, as compared with control group, thus meaning a possible correlation between the increase of EMMPRIN level and an upward risk of NSCLC. Previous studies show that EMMPRIN can induce cancer aggressiveness and angiogenesis via up-regulating the expressions of vascular endothelial growth factor (VEGF) and epidermal growth factor receptor (EGFR), and promote invasion and metastasis by the up-regulation of matrix metalloprotease (MMP) [19]. In addition, elevated EMMPRIN expression in tumor tissues was correlated with shorter overall survival and disease free survival [19]. In our study, NSCLC with the increased EMMPRIN level in serum of NSCLC patients seems to be correlated with malignant phenotype of NSCLC such as lymph node metastasis, poorly differentiated tissues and advanced stage of NSCLC patients. Our study showed that the higher the degree of tumor progression is developed, the higher the serum expression of EMMPRIN is showed, suggesting that EMMPRIN increased with tumor invasion and invasion. When the serum test of EMMPRIN was used to discern NSCLC patients from control group, it responded a better screening ability with a sensitivity of 97.22% and specificity of 95%, suggesting that highly expressed EMMPRIN may be an indicator of NSCLC diagnosis and judgment of invasive degree. We found that a threshold of 80.3 pg/mL of EMMPRIN could discern the NSCLC patients theoretically from control group, which indicated that EMMPRIN can be applied to distinguish NSCLC. Previous research reports that the high expression of EMMPRIN in primary colorectal adenocarcinomas is an important prognostic factor, and patients with EMMPRIN-negative tumours had a relatively good prognosis [20]. In addition, increased expression of EMMPRIN may enhance gastric cancer growth, invasion and angiogenesis by up-regulating MMP expression, and EMMPRIN is considered to be an objective and effective marker for predicting invasion and prognosis [18].

In our study, serum EMMPRIN level was significantly higher in NSCLC patients than in control group and serum EMMPRIN level reflects the development and metastasis of NSCLC. Especially, the statistical analysis suggested that there was a positive expression correlation in NSCLC tissues and serum. The risk ratio analysis also indicated that the up-regulation of EMMPRIN might be an unfavorable factor in NSCLC. The risk ratio values of EMMPRIN higher expression for NSCLC in serum and tissues were 1.56× and 1.1×, respectively. These results suggest that EMMPRIN expression levels are of significance in the diagnosis and prediction of NSCLC. However, before the EMMPRIN is considered as a tumor marker for NSCLC, a larger sample of clinical studies should be required.

Although we have done our best, several deficiencies existed in this study. Firstly, the selection of surgical specimens may result in a selection bias of patients because surgery is involved only in patients with stage IIIB and below. Secondly, the number of research samples was not large, thus larger samples research of NSCLC patients is needed to get a high level of evidence. Thirdly, we did not explore the signal mechanism of EMMPRIN. Hence, further molecular biology experiments on EMMPRIN in NSCLC should be performed to explain the mutual regulation-mechanism regarding lung cancer cell lines.

## Conclusions

We demonstrated that EMMPRIN was upregulated in tissues and serum of NSCLC, and the upregulation of EMMPRIN in NSCLC was associated with poorly differentiated NSCLC, lymph node metastasis and advanced stage of NSCLC patients. In addition, the serum EMMPRIN could serve as a potential biomarker for discerning NSCLC patients. These results indicated that the up-regulation of EMMPRIN was potentially involved in the progression and prognosis of NSCLC.

## Abbreviations

CD147: Cluster of differentiation 147
CEA: Carcinoembryonic antigen
CYFRA 21-1: Cytokeratin 19 fragments
EGFR: Epidermal growth factor receptor
ELISA: Enzyme-linked immunosorbent assay
HRP: Horseradish peroxidase
IHC: Immunohistochemistry
LAC: Lung adenocarcinoma
LSCC: Lung squamous cell carcinoma
MMP: Matrix metalloprotease
NSCLC: Non-small cell lung cancer
PBS: Phosphate buffer saline
ROC: Receiver operating characteristic
TMA: Tissue microarray
TMB: 3,3′,5,5′-Tetramethylbenzidine
TNM: Tumor-node-metastasis
VEGF: Vascular endothelial growth factor
